# Giant epidermoid cyst: a rarity or negligence?

**DOI:** 10.11604/pamj.2018.30.237.15647

**Published:** 2018-07-31

**Authors:** Rakesh Sharma, Biren Padhy

**Affiliations:** 1 Department of Surgery, Institute of Medical Science, Sum Hospital, Bhubaneswar, India

**Keywords:** Epidermoid, giant, gluteal

## Image in medicine

A 35 year old male presented to our outpatient department with a slow growing swelling over the left gluteal region for more than 10 years. There was no history of pain, tenderness, fever or previous trauma. On examination there was a 10 cm x 10cm swelling in the upper outer quadrant of left gluteal region. It was soft, cystic, mobile swelling and adherent to overlying skin with a punctum. Ultrasonography revealed a well-defined hypoechoic heterogenous cyst suggestive of an inclusion epidermoid cyst (A). FNAC was done for confirmation. A complete excision of the whole cyst (B) was done with uneventful post-operative recovery.histopathology showed no signs of malignancy. Epidermoid cyst is one of the commonest benign lesions of skin. Size may vary from a few millimetres to few centimetres but cysts more than 5 cm are known as giant epidermoid cysts and are rare clinical occurrence. Giant epidermoid cyst (A) up to 17 cm in greatest dimension have also been reported. They are slow growing lesions and patients tend to neglect them only to present at a later time. These cysts are usually seen over gluteal region but may be seen over the extremities and scalp. Malignant transformation is a known complication leading to squamous cell carcinoma, basal cell carcinoma or melanomas (C). Clinical examination, USG, FNAC will clinch the diagnosis in most cases. MRI gives additional information in cases of atypical or suspected malignant lesions. Complete excision is the treatment of choice.

**Figure 1:**
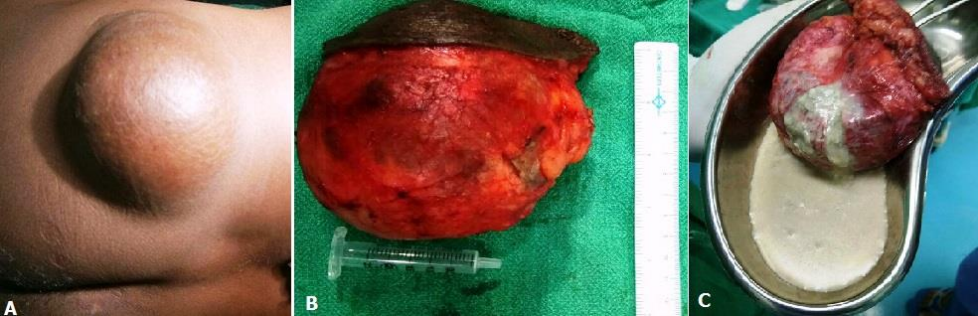
A) giant epidermoid cyst in gluteal region; B) completely excised cyst; C) contents of the cyst

